# Biofilm colonization and succession in a full-scale partial nitritation-anammox moving bed biofilm reactor

**DOI:** 10.1186/s40168-024-01762-8

**Published:** 2024-03-12

**Authors:** Carolina Suarez, Tage Rosenqvist, Ivelina Dimitrova, Christopher J. Sedlacek, Oskar Modin, Catherine J. Paul, Malte Hermansson, Frank Persson

**Affiliations:** 1 Division of Water Resources Engineering, Faculty of Engineering LTH, Lund University, Lund, Sweden; 2 Department of Chemistry and Molecular Biology, University of Gothenburg, Gothenburg, Sweden; 3 Division of Applied Microbiology, Department of Chemistry, Lund University, Lund, Sweden; 4 VA SYD, P.O. Box 191, 20121 Malmö, Sweden; 5 Division of Microbial Ecology, Centre for Microbiology and Environmental Systems Science, University of Vienna, Vienna, Austria; 6 Division of Water Environment Technology, Department of Architecture and Civil Engineering, Chalmers University of Technology, Gothenburg, Sweden

**Keywords:** Wastewater, Anammox, Biofilm, Ecology, Sidestream

## Abstract

**Background:**

Partial nitritation-anammox (PNA) is a biological nitrogen removal process commonly used in wastewater treatment plants for the treatment of warm and nitrogen-rich sludge liquor from anaerobic digestion, often referred to as sidestream wastewater. In these systems, biofilms are frequently used to retain biomass with aerobic ammonia-oxidizing bacteria (AOB) and anammox bacteria, which together convert ammonium to nitrogen gas. Little is known about how these biofilm communities develop, and whether knowledge about the assembly of biofilms in natural communities can be applied to PNA biofilms.

**Results:**

We followed the start-up of a full-scale PNA moving bed biofilm reactor for 175 days using shotgun metagenomics. Environmental filtering likely restricted initial biofilm colonization, resulting in low phylogenetic diversity, with the initial microbial community comprised mainly of *Proteobacteria*. Facilitative priority effects allowed further biofilm colonization, with the growth of initial aerobic colonizers promoting the arrival and growth of anaerobic taxa like methanogens and anammox bacteria. Among the early colonizers were known ‘oligotrophic’ ammonia oxidizers including comammox *Nitrospira* and *Nitrosomonas* cluster 6a AOB. Increasing the nitrogen load in the bioreactor allowed colonization by ‘copiotrophic’ *Nitrosomonas* cluster 7 AOB and resulted in the exclusion of the initial ammonia- and nitrite oxidizers.

**Conclusions:**

We show that complex dynamic processes occur in PNA microbial communities before a stable bioreactor process is achieved. The results of this study not only contribute to our knowledge about biofilm assembly and PNA bioreactor start-up but could also help guide strategies for the successful implementation of PNA bioreactors.

Video Abstract

**Supplementary Information:**

The online version contains supplementary material available at 10.1186/s40168-024-01762-8.

## Background

Reactive nitrogen species, which are environmental pollutants present in wastewater, can be converted into harmless nitrogen gas by microbial communities through biological nitrogen removal (N-removal) processes in wastewater treatment plants (WWTPs). Since microbial communities in WWTPs perform designed and measurable microbial processes, these systems lend themselves well to the study of the intersection between theoretical and applied microbial ecology [1] as colonization, assembly, and succession within microbial communities can be linked to desired or undesired microbial processes.

For N-removal processes in WWTPs, biomass retention systems such as granular bioreactors, moving bed biofilm reactors (MBBRs), integrated fixed film activated sludge (IFAS), rotating biological contactors, membrane bioreactors, and trickling filters are commonly used [2–4]. These types of systems allow for the formation and preservation of stable microbial communities over time, while enabling the retention of essential but slow-growing autotrophic bacteria such as aerobic ammonia-oxidizing bacteria (AOB) and bacteria capable of anaerobic ammonia oxidation (anammox). The physical structures of these biofilms offer protection against predation and environmental stress while creating redox gradients that lead to the coexistence of multiple ecological microniches [5].

One-stage partial nitritation anammox (PNA) biofilm reactors are a type of biomass retention system where both aerobic and anaerobic ammonia oxidation are required to function in tandem for N-removal [6]. Here, AOB grow in the outermost biofilm layers producing nitrite and consuming oxygen. Anammox bacteria in the deeper anoxic layers of the biofilm then convert this nitrite together with free ammonia to harmless nitrogen gas [6, 7]. PNA bioreactors are commonly used to treat sludge liquor from anaerobic digestion (i.e., sidestream water), while implementation of PNA for treating municipal wastewater (i.e., mainstream water) has been more difficult [8].

Although PNA biofilms are often described in terms of their aerobic and anaerobic ammonia-oxidizing populations, molecular approaches like fluorescent in situ hybridization (FISH), amplicon sequencing, and metagenomics have highlighted the diversity of PNA biofilm microbial communities [9–12]. For instance, nitrite-oxidizing bacteria (NOB), whose presence is undesired because they compete with the anammox bacteria for nitrite, are often observed [7]. Other bacterial taxa, such as diverse members of the phyla *Chloroflexota* and *Proteobacteria* are also often present [13] and have potential roles in biofilm architecture, carbon cycling, and denitrification [11]. Eukaryotes have been shown to colonize PNA biofilms and some actively predate on AOB and anammox bacteria [14]. A greater understanding of how these complex PNA biofilm communities form, mature, and change with time is needed to ensure the quick start-up of new PNA bioreactors and prevent bioreactor failures.

Studies of other multispecies biofilms in natural and engineered environments [15–19] have contributed to our knowledge about colonization, succession, and assembly of biofilm communities. However, it is unclear how ecological processes are involved in the development of PNA biofilm communities. Specifically, little is known about the colonization process in PNA reactors seeded only with the incoming wastewater, as anammox and PNA bioreactors are often pre-seeded with biomass from other, well-functioning reactors to reduce initial reactor start-up time [20–22].

In this study, we investigated the start-up of a full-scale one-stage PNA MBBR at the Klagshamn WWTP (Malmö, Sweden) treating sidestream wastewater. Notably, this PNA MBBR achieved stable N-removal after only 180 days, despite only being seeded by influent wastewater [23]. Throughout the 180-day start-up period, biofilm samples were taken frequently and shotgun metagenomics was used to resolve metagenome-assembled genomes (MAGs) of bacteria, archaea, and eukaryotes in this system. The aim was to capture the colonization and succession of the multispecies microbial biofilm communities and to link observed changes to bioreactor performance. As stable N-removal was the goal of this PNA reactor, the population dynamics of N-converting bacteria (AOB, NOB, and anammox bacteria) in relation to reactor performance were of particular interest.

## Material and methods

A bioreactor at the Klagshamn WWTP (Malmö, Sweden) was fed with sludge liquor from anaerobic digestion of primary and secondary sludge. The start-up of this bioreactor is described in detail elsewhere [23]. Briefly, an MBBR with a volume of 256 m^3^ was filled to 40% with pristine biofilm K5 carriers (Veolia Water Technologies AB–AnoxKaldnes, Lund, Sweden), and operated at a temperature of ∼ 30 °C. The carriers were kept for one week in pre-precipitated and pre-settled wastewater (PSW) until bioreactor operation began. The nitrogen load was slowly increased with time by increasing the fraction of sludge liquor in the feed. Pristine carriers were submerged in PSW on December 11, 2018, and bioreactor operation began on December 18, 2018 (henceforth referred to as day 1 using the same numbering of days as in Dimitrova et al. 2020). Reactor conditions during the start-up are summarized in Figure S1.

### Sampling and DNA extraction

Weekly samples were taken for four weeks after bioreactor start-up (days 1, 10, 17, and 22), followed by bi-weekly samples for the rest of the 181-day start-up period (days 37, 51, 63, 78, 92, 107, 120, 134, 148, 162, and 175). On each sampling date, three K5 biofilm carriers were removed from the reactor and stored at -20 °C until DNA was extracted. Samples from all fifteen sampling dates were sequenced, for a total of 45 samples. A FastDNA SPIN kit for soil (MP Biomedicals, Santa Ana, CA, USA) was used for DNA extraction as previously reported [24]. The biofilm was removed from individual K5 carriers by brushing it into 4 ml of sterile water, with the resulting suspension being transferred to a 15-ml centrifuge tube. The suspension was centrifuged at 4653 g for 3 min and the supernatant was discarded. Nine hundred seventy-eight microliters of sodium phosphate buffer and 122 μl of MT buffer, from the FastDNA SPIN kit for soil, were added to the 15 ml centrifuge tubes. The biofilms were resuspended by pipetting and 1.1 ml of the suspensions were transferred to Lysing Matrix E tubes. FastPrep homogenization and subsequent purification steps were done according to manufacturer instructions. Total DNA concentration was determined with a Qubit 3.0 fluorometer using the dsDNA High Sensitivity kit (Thermo Fisher Scientific, USA).

### Sequencing and assembly

Forty-five libraries were prepared with a TruSeq PCR-free kit (Illumina) and were sequenced on NovaSeq6000 with a 2 × 151 setup, resulting in a 505-Gbp metagenome.

Low-quality reads and adapters were removed with fastp [25]. Metagenomic assembly of contigs was done in Megahit v1.29 using default settings, except for a minimum contig length of 1000 bp [26]. To facilitate metagenome assembly, assembly was done for only 15 samples, by choosing one of three K5 biofilm carrier replicates of each sampling date, while all 45 samples were used for the estimation of MAG coverage and relative abundance. Due to the potential of strain variation across the time series, individual assemblies were done, instead of a co-assembly. This resulted in 15 assemblies containing between 208 and 920 Mbp, and between 70,000 and 300,000 contigs.

### Eukaryotic and prokaryotic MAGs

Prior to binning, reads from the 15 selected samples were mapped to the respective assemblies using Bowtie2 v2.3 with default settings [27]. To recover potential eukaryote MAGs, the approach by West et al. [28] was used, where eukaryote contigs are filtered by their k-mer frequencies. Contigs from the 15 assemblies were prefiltered with eukrep [28] into eukaryotic and prokaryotic fractions using default settings. Binning and gene prediction of eukaryotic MAGs are described in detail in the supporting information.

Binning of the individual prokaryotic assemblies was performed with MetaBAT2 with default settings [29] followed by genome dereplication in dRep [30] using a 98% average nucleotide identity (ANI) threshold. Ten percent contamination criteria and the default of 75% completeness were used for filtering with CheckM [31]. Gene prediction in prokaryotes was done with prodigal v2.6.3 in normal mode [32]. Taxonomic assignment of prokaryotic MAGs was done with GTDB-Tk v2.1.0 [33], using the GTDB r207 taxonomy [34, 35] (Supplementary dataset S1). ANI was estimated with fastANI [36]. The KLAP57 MAG, classified as a complete ammonia oxidizer (comammox) *Nitrospira*, had high completeness (95.9%) and low contamination (7.73%), but a genome size of 5.7 Mb which is substantially larger than other comammox genomes [37]. It was therefore manually refined using Anvi'o [38] resulting in a 3.6 Mb MAG with 94.9% completeness and 4.09% contamination.

### Identification of aerobes and anaerobes

Functional annotation of the MAGs was performed using eggNOG-mapper v2.1.9 [39] based on eggNOG orthology data [40]. Sequence searches were performed using DIAMOND [41]. Five oxygen reductases were used as potential markers for an aerobic lifestyle, cytochrome c oxidase (*coxABC*), cytochrome c oxidase cbb3-type (*cooNOPQ*), cytochrome bd oxidases (*cydAB*) cytochrome o ubiquinol oxidases (*cyoABCDE*), and the eukaryotic cytochrome c oxidase (*cox1*).

### Statistics

Bowtie2 was used to build an index database of all MAGs, which was used for mapping reads across all 45 samples. This was used to estimate the relative abundance of MAGs in all 45 samples with coverM v0.4.0 (https://wwood.github.io/CoverM). To assess assembly mechanisms, a phylogenetic tree of all bacterial MAGs was constructed with GtoTree v1.7.00 [42] using concatenated gene alignments of 72 single-copy genes. The bacterial phylogenetic tree was used to estimate Faith´s phylogenetic diversity (PD) [43], which is sensitive to terminal clustering of the phylogenetic tree [44]. PD often correlates with species richness [45], which was also observed in this study. As such, the standardized effect size of PD (PD_SES_) was calculated, where PD values are compared against the randomized values of a null model. The standardized effect size of the weighted mean nearest taxon distance (MNTD_SES_) was also estimated [46]. Both PD_SES_ and MNTD_SES_ were calculated in R. 4.3.1 [47] using the R-package Picante v1.8.2 [48] with the functions ses.pd and ses.mntd using 999 permutations and the null model “taxa.labels”. Low PD_SES_ and MNTD_SES_ values indicate phylogenetic clustering (i.e., a community that is more closely related than would be expected by chance), while high values indicate phylogenetic overdispersion (i.e., a community that is less closely related than would be expected by chance).

For nitrifier- and anammox MAGs, a redundancy analysis (RDA) [49] was done with the R-package Vegan v2.6.4 [50] using the rda function, after doing a Hellinger transformation of the community abundances using the decostand function to lessen the importance of highly abundant taxa. Possible explanatory variables for the RDA were chosen with forward selection [51] using the ordiR2step function in Vegan and a stopping significance level of 0.01.

### Read-based analysis

As a complement to the MAG-based approach of taxonomic profiling, a read-based analysis was done using MetaPhLan v4.1.0 [52]. Reads were analyzed using default settings and the database mpa_vOct22_CHOCOPhlAnSGB_202212. The fraction of unclassified reads was on average 85%, ranging between 78 and 94%

## Results

### Biofilm carriers were rapidly colonized by Proteobacteria

**Fig. 1:**
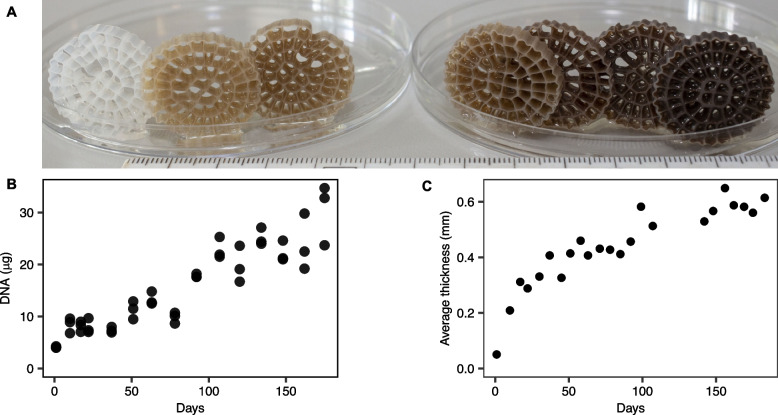
Biofilm carrier colonization over time. **A** Representative individual K5 biofilm carriers (from left to right, day 1, 22, 51, 78, 107, 134, and 162); the ruler shows the size in cm. **B** Amount of extracted DNA from individual K5 biomass carriers. DNA was extracted from three K5 carriers at each sampling timepoint. **C** Biofilm thickness over time

Visual observations showed that the biofilm on the plastic carriers became thicker and darker with time (Fig. 1A). The amount of extracted DNA per biofilm carrier increased with time (Pearson, *r* = 0.87, *p* < 0.01) (Fig. 1B). A positive correlation between extracted DNA (ng/mm^2^) (this study) and biomass on the biofilm carriers (gDS/m^2^) [23] was observed (Pearson, *r* = 0.73, *p* < 0.01). Biofilm thickness also increased over time reaching 0.6 mm (Fig. 1C) by day 150.

**Fig. 2:**
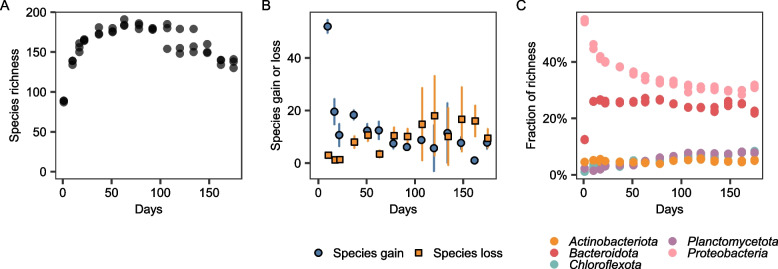
Alpha diversity over time of the biofilm communities. **A** Species richness. **B** Species gain and species loss when comparing a time point with the previous one; points show averages and bars show standard deviations. **C** Fraction of species richness for major bacterial phyla representing > 5% of the total richness at any given timepoint

After dereplication, a total of 213 bacterial MAGs, five archaeal MAGs, and six eukaryote MAGs were resolved. The coverage of the genomes in terms of relative abundance was used as a proxy for species abundance, while species richness was estimated by counting the number of MAGs with a relative abundance above zero. Throughout the whole 180-day start-up period, the amount of biomass and DNA on the K5 carriers continued to increase (Fig. 1B), but species richness did not (Fig. 2A). On day 1 of the bioreactor operation, species richness was 89.3 ± 1.2 (average ± standard deviation), indicating a rapid colonization of the biofilm carriers. The highest richness was observed on day 63 (186 ± 4.4), and then decreased, reaching 136.3 ± 5.7 at day 175 (Fig. 2A). A similar trend of species richness decreasing with time was observed with a read-based analysis (Figure S6). Both species-gain and species-loss processes co-occurred over the 180-day period, with species-gain initially dominating, while later, species-loss became more important (Fig. 2B). Despite these gain and loss processes, around half of the species (40.7 ± 2.8) observed on day 1 were still present at day 175.

The initial community was largely dominated by *Proteobacteria* with 48 to 49 MAGs, which represented around 55% of the total richness at day 1 (Fig. 2C). At day 1, the *Proteobacteria* community was largely dominated by *Burkholderiaceae* (32–34% relative abundance), including the genera *Rhodoferax*, *Aquabacterium,* and *Rubrivivax*. The number of *Proteobacteria* MAGs increased until day 17, reaching 63 to 66 MAGs. However, the *Proteobacteria* comprised a smaller amount of the total richness over time, settling at around 30%. On day 1, *Bacteroidota* represented around 12% of the total richness, after which it rapidly increased to 26% on day 10 and then remained relatively constant throughout the entire study (Fig. 2C). Two to three *Planctomycetota* and one to two *Chloroflexota* MAGs were observed on day 1, which steadily increased to 10-11 and 11 MAGs respectively by day 175.

### Aerobes preceded the arrival of anaerobes during biofilm development

**Fig. 3:**
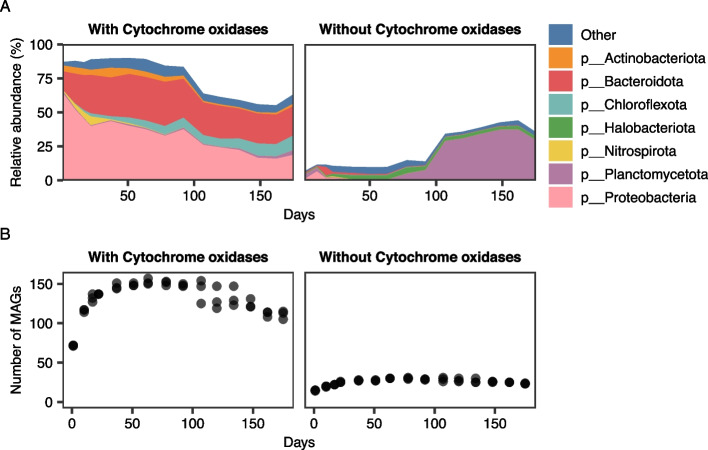
Bacterial and archaeal MAGs with and without terminal oxidases. **A** Relative abundance of MAGs classified at the phylum level, showing average values of the three replicates at each sampling occasion. **B** Number of MAGs

For eukaryotic MAGs, *COX1* genes could not be detected, but this could have been due to a failure to recover mitochondrial sequences [53]. Out of 218 MAGs in the Prokaryotic community, 183 had identifiable aerobic terminal oxidase genes and were largely affiliated with the *Proteobacteria* or *Bacteroidota* (Fig. 3A, Figure S2). These putatively aerobic microorganisms dominated the initial biofilm, with their relative abundance from day 1 to day 92 ranging between 83 and 89% (Fig. 3A). A decline in their relative abundance was observed after day 92, reaching a low of 55% on day 162. (Fig. 3A). In contrast, the relative abundance of 35 prokaryotic MAGs lacking terminal oxidases increased with time (Fig. 3B). They were present throughout the entire study, with 15 to 16 MAGs on day 1, later increasing to around 31 MAGs on day 63 (Fig. 3B).

Although some MAGs may be lacking cytochrome oxidases due to incompleteness, several MAGs without cytochrome oxidases were affiliated with taxa where an anaerobic lifestyle has been previously observed or inferred. These include three anammox bacteria *Ca*. Brocadia MAGs [54], five archaea MAGs in the order *Methanomicrobiales*, KLAP108 and KLAP91 in the phylum *Thermotogota* [55], KLAP90 in the phylum *Ca*. Cloacimonadota [56], KLAP100 in the class *Clostridia* [57] and KLAP102 in the family *Syntrophorhabdaceae* [58]. In the phylum *Patescibacteria,* terminal oxidases are absent [59] and this was also observed for the five *Patescibacteria* MAGs found in the current study. Additional genomic evidence for the presence of anaerobes in the later stages of the reactor operation was provided by the presence of complete or near-complete pathways for methanogenesis within the *Methanomicrobiales* MAGs. The relative abundance of these archaea was 0.29 ± 0.02% on day 1 and reached a maximum of 4.37 ± 0.33% on day 78, before decreasing to 2.55 ± 0.27% on day 175.

### Aerobes and anaerobes had different trends in phylogenetic diversity

Estimating how phylogenetic diversity changes across time could provide further insights into the assembly mechanisms of the PNA microbial community. In addition, to examine if assembly processes of anaerobic and aerobic bacteria might differ in this context, phylogenetic diversity was also estimated separately for both groups.

**Fig. 4:**
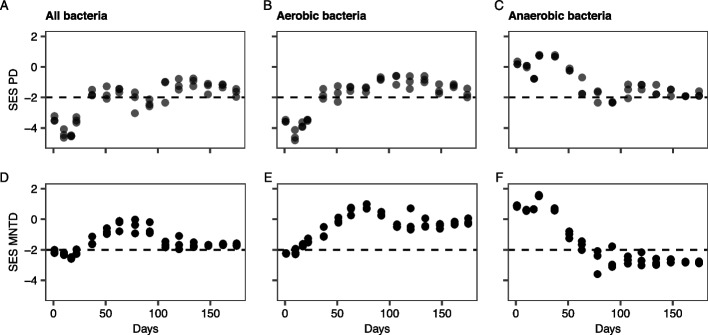
Phylogenetic diversity. **A**,** D** All bacteria. **B**,** E** Putative aerobic bacteria. **C**,** F**: Putative anaerobic bacteria. **A**–**C** The standardized effect size of Faith´s phylogenetic diversity (PD_SES_). **D**–**F** The weighted mean nearest taxon distance (MNTD_SES_). The dashed horizontal line represents − 2 SES; for the samples with SES below − 2, SES is significantly lower than expected by random chance

Both the standardized effect size of Faith diversity (PD_SES_) and the MNTD_SES_ showed that phylogenetic clustering occurred during the first stages of biofilm formation, until day 22 among bacteria (PD_SES_ and MNTD_SES_ <  − 2; Fig. 4A, D), suggesting that the initial biofilm colonization was not entirely stochastic and was carried out by closely related taxa. Notably, for all bacteria, the MNTD_SES_ was positively correlated with the free ammonia (NH_3_) (Pearson, *r* = 0.56,* p* < 0.001), NH_4_^+^ (Pearson, *r* = 0.50, *p* < 0.001), and NO_2_^−^ concentrations (Pearson, *r* = 0.54, *p* < 0.001) (Table S1).

For putative aerobic bacteria, phylogenetic diversity trends across time were similar to that of the overall community (Fig. 4B, E) with less clustering after the initial biofilm formation. Interestingly, the putative anaerobic microorganisms showed the opposite pattern with decreasing phylogenetic diversity over time and phylogenetic clustering being observed from day 78 onwards (Fig. 4C, F).

### Comammox bacteria and cluster 6a AOB were replaced by cluster 7 AOB

**Fig. 5:**
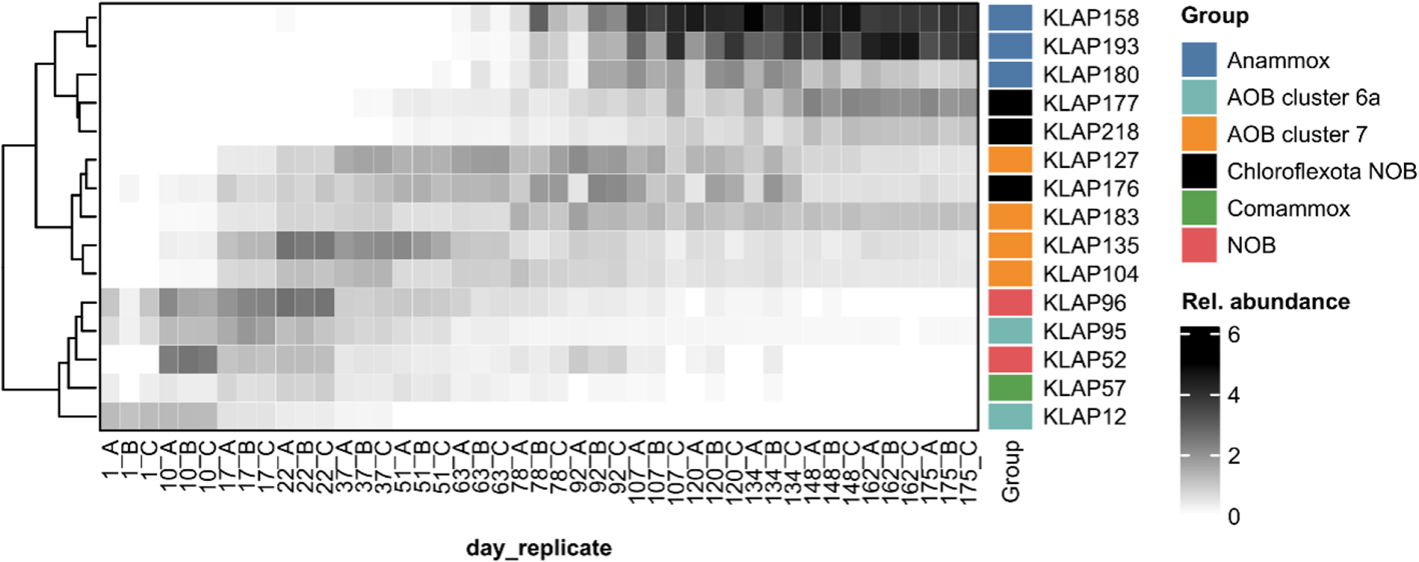
Heatmap showing the relative abundance (%) of anammox bacteria, nitrifiers, and putative NOB within *Chloroflexota*. Rows (MAGs) are clustered by hierarchical clustering of Bray–Curtis distances. Sample names indicate the sampling day and sample replicate

Aerobic ammonia oxidizers within the genera *Nitrosomonas and Nitrospira* were detected throughout the entire start-up process and exhibited several shifts and succession events over the course of the 175-day reactor start-up (Fig. 5). Ammonia-oxidizing colonizers were detected on day 1 and consisted of two *Nitrosomonas* cluster 6a AOB MAGs (KLAP12 and KLAP95) (Figure S3) and one complete ammonia oxidizer (comammox) MAG (KLAP57). KLAP57 shares 94.6% ANI with the known comammox *Nitrospira nitrosa* [60] and contains a gene cluster encoding both hydroxylamine oxidoreductase subunits, cytochrome *c*_554_, and cytochrome *c*_m552_ as well as several copies of the ammonia monooxygenase subunit c (*amoC*).

All three colonizing ammonia oxidizers (KLAP12, KLAP57, and KLAP95) achieved their peak relative abundance on day 17 (3.51 ± 0.52%). However, by day 17, an additional four *Nitrosomonas* Cluster 7 AOB MAGs (KLAP104, KLAP127, KLAP135, and KLAP183) (Figure S3) were also detectable in the biofilm. The comammox *Nitrospira* and the two *Nitrosomonas* cluster 6a AOB populations were replaced over time by the *Nitrosomonas* Cluster 7 AOB. In contrast to the colonizing ammonia oxidizers, which became undetectable or almost undetectable (0.017 ± 0.002% relative abundance) by the end of the 175-day start-up period, all four *Nitrosomonas* cluster 7 AOB populations remained throughout the rest of the reactor start-up (Fig. 5, Figure S4). Read-based analysis also indicated a replacement of cluster 6a *Nitrosomonas* (*Nitrosomonas oligotropha*, *Nitrosomonas* sp. JL21, and *Nitrosomonas ureae*) by cluster 7 *Nitrosomonas* (*Nitrosomonas europaea*) (Figure S7).

### NOB were transient members of the biofilm

At least two NOB MAGs were detected during the initial colonization phase of the biomass carriers; KLAP96, which shares 96.6% ANI with *Nitrospira* ND1 [61], and KLAP52 which shares 98.6% ANI with *Ca*. Nitrotoga fabula [62]. Both MAGs peaked in relative abundance on day 22 and were undetectable by day 148 (Fig. 5, Figure S4). Unlike the population succession observed with the AOB, the initial colonizing NOB was not replaced with other canonical NOB on the biofilm carriers, as no *Nitrospira* or *Ca.* Nitrotoga MAGs were detected at the end of the study (day 175). Notably, three putative NOB MAGs within the *Chloroflexota*, with a *nxrA/narG* similar to that of the NOBs *Nitrobacter* and *Nitrolancea* were observed, KLAP176, KLAP177, and KLAP218 (Figure S5). Their dynamics differed from that of the *Nitrospira* and *Ca*. Nitrotoga, as they increased in relative abundance as *Nitrospira* and *Ca.* Nitrotoga was decreasing (Fig. 5, Figure S4). The read-based analysis also showed the presence of NOB (multiple species of *Nitrospira* as well as *Ca.* Nitrotoga fabula) in the early stages of colonization, which were not detectable after day 148 (Figure S7).

### Anammox bacteria were late colonizers

Three anammox bacteria MAGs were observed in the PNA biofilms, KLAP158, KLAP180, and KLAP193 classified as *Ca*. Brocadia pituitae, *Ca*. Brocadia sapporoensis and *Ca*. Brocadia fulgida respectively. They were often below the detection limit until day 63. When summed together their abundance was 0.2 ± 0.2% on day 63 but rapidly increased to 28.4 ± 9.8% on day 107, and reached 36.7 ± 3.5% on day 148 (Fig. 5, Figure S4). KLAP180 reached a maximum of around 3.2 ± 1.3% at day 134, and it decreased afterward. In contrast, the abundance of KLAP158 and KLAP193 continued to increase, with both becoming not only the dominant anammox bacteria at the end of the study but also the two most abundant species in the biofilm (Fig. 5, Figure S4). Only *Ca.* Brocadia sapporoensis was identified by the read-based analysis. As with KLAP180, it was below the detection limit until day 63 and reached its maximum relative abundance at around day 134 (Figure S7).

### The abundance of ammonia and nitrite oxidizers was linked to reactor conditions

**Fig. 6:**
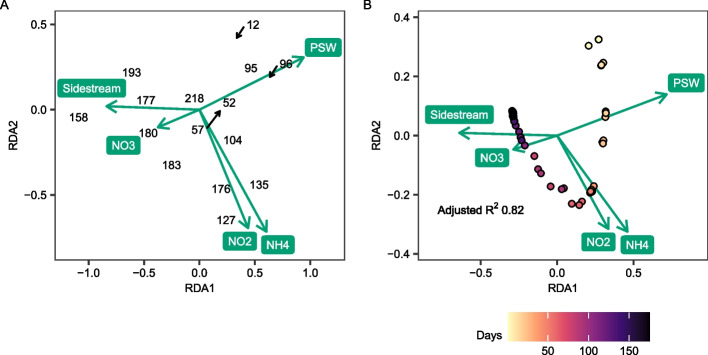
RDA biplot of Hellinger transformed data of nitrifiers, anammox bacteria, and putative NOB within the *Chloroflexota*. **A** MAGs. **B** Samples. To avoid overlap of labels, only the MAG numbers without the prefix KLAP are shown, and for some MAGs labels have been moved, with their original position in the plot indicated by black arrows

RDA was used to identify reactor conditions that correlated with the relative abundance of aerobic ammonia oxidizers, nitrite oxidizers, and anammox bacteria detected on the biofilm carriers. PSW flow, sidestream flow, and the concentration of NH_4_^+^, NO_2_^−^, and NO_3_^−^ explained 82% of the variation of these taxa (Fig. 6). For the NOB *Ca*. Nitrotoga KLAP52, *Nitrospira* KLAP96, and the AOB cluster 6a *Nitrosomonas* KLAP95 and KLAP12, which were all more abundant at the beginning of the start-up, there was a correlation between the PSW flow to the reactor and their relative abundance. The concentration of NH_4_^+^ and NO_2_^−^ in the reactor was linked to the relative abundance of all four *Nitrosomonas* cluster 7 AOB KLAP135, KLAP127, KLAP183, and KLAP104. Finally, sidestream flow and the nitrite concentration in the reactor were correlated to the relative abundance of the anammox *Ca*. Brocadia KLAP158 and KLAP193 MAGs (Fig. 6a).

## Discussion

The observed changes in alpha diversity over time in the PNA bioreactor biofilm community, with alpha diversity rapidly increasing at the beginning and then decreasing with time (Fig. 2), are similar to those previously seen in lake biofilms [16, 17], drinking water biofilms [19], river biofilms [18], and groundwater sediments [63]. This suggests that the microbial assembly processes of other natural and anthropogenic biofilms also occur in PNA communities.

Biofilm formation occurs in three major steps: aggregation and attachment, growth and accumulation, and disaggregation and detachment [64]. The initial biofilm attachment has been suggested to be a largely stochastic process with limited selection pressure [17, 19]. However, in this study initial biofilm colonization was not entirely stochastic, as phylogenetic clustering occurred during the first stages of biofilm formation (Fig. 4). The initial community was largely dominated by *Proteobacteria*, supporting the idea that the initial biofilm colonization was not completely random. Initial biofilm colonization by *Proteobacteria* has been shown in other multispecies biofilms: in rivers [18], marine settings [65, 66], and on reverse osmosis membranes [67], suggesting that *Proteobacteria* are better adapted to initial biofilm attachment than other microorganisms. Although generalized traits that would result in *Proteobacteria* being pioneers for biofilm formation are not identified, most *Proteobacteria* and *Bacteroidota* that colonized the PNA biofilms in this study had the ability to use oxygen as an electron acceptor indicating that the initial colonization observed was carried out by aerobes.

As the biofilm matures, the number of available niches within the biofilm environment will also increase [68]. Air was supplied to the bioreactor, preventing initial biofilm colonization by strict anaerobes (Figs. 3 and 4), but biofilm growth by aerobic bacteria results in the formation of oxygen gradients and anaerobic regions within the biofilm structure itself [69]. In the oxic environment of a PNA bioreactor, prior biofilm growth would thus favor the establishment of anammox bacteria [70]. This has been previously shown for nitrifying biofilms, where anammox bacteria were virtually absent from thin aerobic biofilms, while their abundance was higher in thicker biofilms [24]. The current study shows that the observed increase in abundance of methanogens, anammox bacteria, and other anaerobes after ∼ 100 days (Figs. 3 and 5) might be in part due to facilitative priority effects [71]: the initial establishment of pioneer aerobes and subsequent creation of anoxic regions prepared new ecological niches that allowed obligate anaerobes to survive in the biofilm. As anammox bacteria use ammonium and NO_2_^−^ as substrates, prior production of NO_2_^−^ by AOB would also facilitate their growth. Future studies using dissolved oxygen microsensors to follow the development of the oxygen gradient as the biofilm thickness increases would provide quantitative insights into the formation of functional PNA biofilms.

In general, facilitation is expected to cause phylogenetic overdispersion [72]. In contrast, phylogenetic clustering was observed in this study, first for aerobic microorganisms, and later for anaerobic microorganisms. The observed phylogenetic clustering could be due to environmental filtering [46, 73] caused by the reactor operation. Conditions were not constant throughout this study; to prevent substrate inhibition during the start-up, the nitrogen load was initially low and increased progressively [23]. Consequently, there was an increase in the reactor concentration of NH_4_^+^ and its un-ionized form NH_3_, which can cause environmental filtering through growth inhibition [74], and by serving as a substrate for ammonia oxidizers [75]. The importance of NH_4_^+^ and NH_3_ in the microbial community is also seen in the positive correlation of MNTD_SES_ with NH_4_^+^ and NH_3_ (Table S1). A correlation between MNTD_SES_ and NO_2_^−^ was observed, which is difficult to disentangle from the effects of NH_3_/NH_4_^+^, as it is continuously transformed into NO_2_^−^ by AOB.

While MTND_SES_ was correlated with the concentrations of nitrogen species in the reactor, PD_SES_ correlates with process parameters (centrate flow, PSW flow, and HRT). As PD is a presence-absence metric, and thus sensitive to the presence of low-abundant taxa, it may reflect immigration from the influents, which would be altered by changing the process parameters. This highlights the potential role of water type in the colonization process of PNA MBBRs.

The sustained, high total ammonium concentration (> 60 mg/l, days ~ 20–100), as PSW flow was decreased and the sludge liquor flow was increased, was likely a driving factor for the shift in the ammonia oxidizer community. It is well documented that *Nitrosomonas* cluster 7 AOB are adapted to higher ammonium concentrations than those tolerated by their more oligotrophic *Nitrosomonas* cluster 6a and comammox *Nitrospira* counterparts [76–78]. Interestingly, once established the *Nitrosomonas* cluster 7 AOB remained in the biofilm, even after free ammonium in the reactor dropped back near initial values. A recent survey of full-scale N-removal bioreactors showed that although comammox *Nitrospira* are common in mainstream N-removal reactors, they are absent in sidestream PNA systems [79]. This study shows that comammox *Nitrospira* can occur in full-scale sidestream PNA systems, but likely only during the initial start-up period if the nitrogen load is low. The observed disappearance of *Nitrospira* could be due to an NH_3_ concentration well above the reported inhibition values for *Nitrospira,* at more than 10 mg-NH_3_/l from day 37 onwards [80, 81]. The use of intermittent aeration may also have contributed to the inhibition of *Nitrospira* [23].

From day 80 to 100, NO_3_^−^ production was higher than was theoretically expected for anammox bacteria [23], which also coincided with an increase in the relative abundance of *Ca*. Nitrotoga (Fig. 5, Figure S4). *Ca.* Nitrotoga seems to be more resistant to NH_3_ than *Nitrospira* [82, 83], and thus NH_3_ inhibition strategies used in PNA reactors might select for this NOB [84]. Even so, the presence of *Ca.* Nitrotoga in a bioreactor operating at 30 °C was surprising since it is considered to be a cold-tolerant NOB [82, 85] and is frequently observed in WWTP reactors treating cold wastewater [83]. *Ca*. Nitrotoga fabula, to which the MAG KLAP52 is highly similar, has an optimum growth temperature between 24 and 28 °C [62], which might explain the presence of KLAP52 in the biofilm. An increase in temperature from 30 to 35 °C after day 95 (Figure S1) could have contributed to the loss of *Ca*. Nitrotoga in the later phase of the study period (Figure S4).

The ability to oxidize nitrite to nitrate is also observed among some bacteria in the phylum *Chloroflexota* [86, 87]. Three *Chloroflexota* MAGs had *nar/nxr* similar to those of the *Ca*. Nitrocaldera robusta and *Nitrolancea hollandica* (Figure S5), and it is tempting to speculate that these *Chloroflexota* would also be capable of nitrite oxidation. In contrast to *Ca*. Nitrotoga, *Chloroflexota* NOB are often observed at high temperatures [87, 88], potentially favouring the replacement of *Ca*. Nitrotoga and *Nitrospira* by such NOB in the later stages of the study period (up to day 175).

## Conclusions

This study shows that is possible to achieve a PNA microbial community with nitrifiers and anammox bacteria in MBBRs, using virgin biofilm carriers and the bioreactor influent as inoculum for the biofilm. It is likely that multiple process influenced the assembly of the microbial communities in the PNA biofilms. Facilitative priority effects by the initial aerobic biofilm community and nitrifiers resulted in anaerobic conditions and the production of nitrite, increasing the available niches to allow the establishment of new taxa, with methanogens and anammox bacteria being among these later colonizers. Although nitrifiers were early colonizers, species replacement was observed among members of the guild, with the final population of nitrifiers being different from the initial one. We show that microbial community succession is a key process for biofilm development in PNA bioreactors, and that complex dynamic processes occur before a stable bioreactor process is achieved. Creating conditions for anammox bacteria to thrive is important, which is favored by the prior arrival of AOB, inhibition of NOB, and biofilm growth; the latter perhaps might be achieved by having some organic material in the feed at the beginning of the start-up.

### Supplementary Information

**Additional file 1: Figure S1.** Conditions in the reactor. Data from Dimitrova *et al* [23]. **Figure S2.** Abundance of MAGs with cytochrome oxidases. **Figure S3.** Average nucleotide identity of *Nitrosomonas* genomes and MAGs from this study. **Figure S4.** Relative abundance of ammonia oxidizers, nitrite oxidizers, putative *Chloroflexota* nitrite oxidizers and anammox bacteria MAGs. **Figure S5.** Phylogenetic tree of DMSO reductase family type II. Putative *nxrA/narG* recovered from MAGs in this study are shown in bold. Red labels are known NOB. Circles show branches with more than 95% support. Periplasmatic nitrate reductase (*napA*) was used as the outgroup. The “put. anammox *narG*” cluster, includes *narG*-like genes similar to the putative *narG* of *Ca*. Jettenia ecosi [16]. **Figure S6.** Changes in the overall microbial community assessed with MetaPhlAn. A: Changes in species richness. B: Changes in relative abundance of major phyla. **Figure S7.** Relative abundance of nitrogen converters (nitrifiers, and anammox bacteria) at the species level, as assessed with Methaplan. **Table S1.** Pearson correlation values between reactor conditions and phylogenetic diversity for all bacteria. Highly significant values (*p*<0.001) are marked with an asterisk (*).

**Additional file 2: Supplementary Dataset S1.** Taxonomic assignment of prokaryotic MAGs.

## Data Availability

Raw reads and MAG sequences have been deposited in the European Nucleotide Archive (ENA) at EMBL-EBI under accession number PRJEB58181 ( https://www.ebi.ac.uk/ena/browser/view/PRJEB58181). Genome annotations are available at Zenodo (DOI: 10.5281/zenodo.7775397). Sample metadata, MAG relative abundance, and taxonomic classification of MAGs are provided in the Supplementary Dataset S1.

## Abbreviations

Anammox: Anaerobic ammonia oxidation
AOB: Aerobic ammonia-oxidizing bacteria
Comammox: Complete ammonia oxidizer
IFAS: Integrated fixed film-activated sludge
FISH: Fluorescent in situ hybridization
MAG: Metagenome assembled genome
MBBRs: Moving bed biofilm reactors
MNTD_SES_: Mean nearest taxon distance
N-removal: Nitrogen removal
NOB: Nitrite-oxidizing bacteria
PD: Faith´s phylogenetic diversity
PD_SES_: The standardized effect size of PD
PNA: Partial nitritation-anammox
PSW: Pre-settled wastewater
RDA: Redundancy analysis
WWTP: Wastewater treatment plants
