# Supplementary material for: Biofilm colonization and succession in a full-scale partial nitritation-anammox moving bed biofilm reactor
Source: Microbiome. 2024 Mar 12;12:51. doi: 10.1186/s40168-024-01762-8 (PMC10935808; doi:10.1186/s40168-024-01762-8)
Supplement: Supplementary file 2 — Additional file 1: Figure S1. Conditions in the reactor. Data from Dimitrova et al [23]. Figure S2. Abundance of MAGs with cytochrome oxidases. Figure S3. Average nucleotide identity of Nitrosomonas genomes and MAGs from this study. Figure S4. Relative abundance of ammonia oxidizers, nitrite oxidizers, putative Chloroflexota nitrite oxidizers and anammox bacteria MAGs. Figure S5. Phylogenetic tree of DMSO reductase family type II. Putative nxrA/narG recovered from MAGs in this study are shown in bold. Red labels are known NOB. Circles show branches with more than 95% support. Periplasmatic nitrate reductase (napA) was used as the outgroup. The “put. anammox narG” cluster, includes narG-like genes similar to the putative narG of Ca. Jettenia ecosi [16]. Figure S6. Changes in the overall microbial community assessed with MetaPhlAn. A: Changes in species richness. B: Changes in relative abundance of major phyla. Figure S7. Relative abundance of nitrogen converters (nitrifiers, and anammox bacteria) at the species level, as assessed with Methaplan. Table S1. Pearson correlation values between reactor conditions and phylogenetic diversity for all bacteria. Highly significant values (p<0.001) are marked with an asterisk (*). [file 40168_2024_1762_MOESM1_ESM.docx]

**Supporting information for Biofilm colonization and succession in a full-scale partial nitritation-anammox moving bed biofilm bioreactor**

Carolina Suarez^1*^, Tage Rosenqvist^2^, Ivelina Dimitrova^3^, Christopher J. Sedlacek^4^, Oskar Modin^5^, Catherine J. Paul^1,2^, Malte Hermansson^6^, Frank Persson^5^

^1^ Division of Water Resources Engineering, Faculty of Engineering LTH, Lund University, Lund, Sweden.

^2^ Division of Applied Microbiology, Department of Chemistry, Lund University, Lund, Sweden.

^3^ VA SYD, P.O. Box 191, SE-20121 Malmö, Sweden.

^4^ University of Vienna, Division of Microbial Ecology, Centre for Microbiology and Environmental Systems Science, Vienna, Austria.

^5^ Division of Water Environment Technology, Department of Architecture and Civil Engineering, Chalmers University of Technology. Gothenburg, Sweden.

^6^ Department of Chemistry and Molecular Biology, University of Gothenburg, Gothenburg, Sweden.

* E-mail correspondence: carolina.suarez@tvrl.lth.se

Contents

[SUPPORTING FIGURES 2](#_Toc149903466)

[SUPPORTING TABLES 8](#_Toc149903467)

[SUPPORTING METHODS 8](#_Toc149903468)

[Eukaryotic MAGs 8](#_Toc149903469)

[Identification of putative *Chloroflexota* NOB 8](#_Toc149903470)

[Estimation of biofilm thickness 9](#_Toc149903471)

[SUPPORTING REFERENCES 9](#_Toc149903472)

# SUPPORTING FIGURES


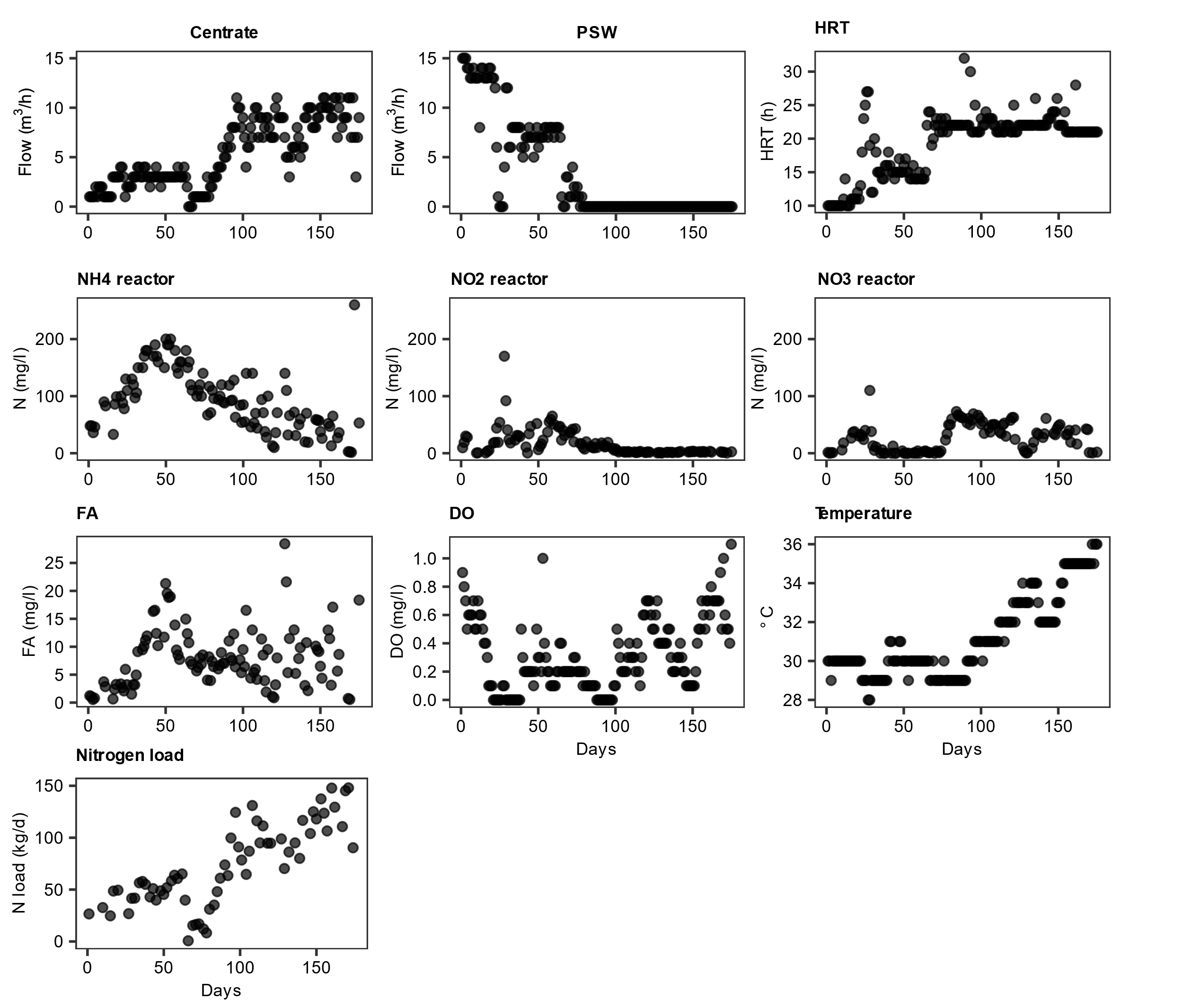


**Figure S1**: Conditions in the reactor. Data from Dimitrova *et al* (2020)


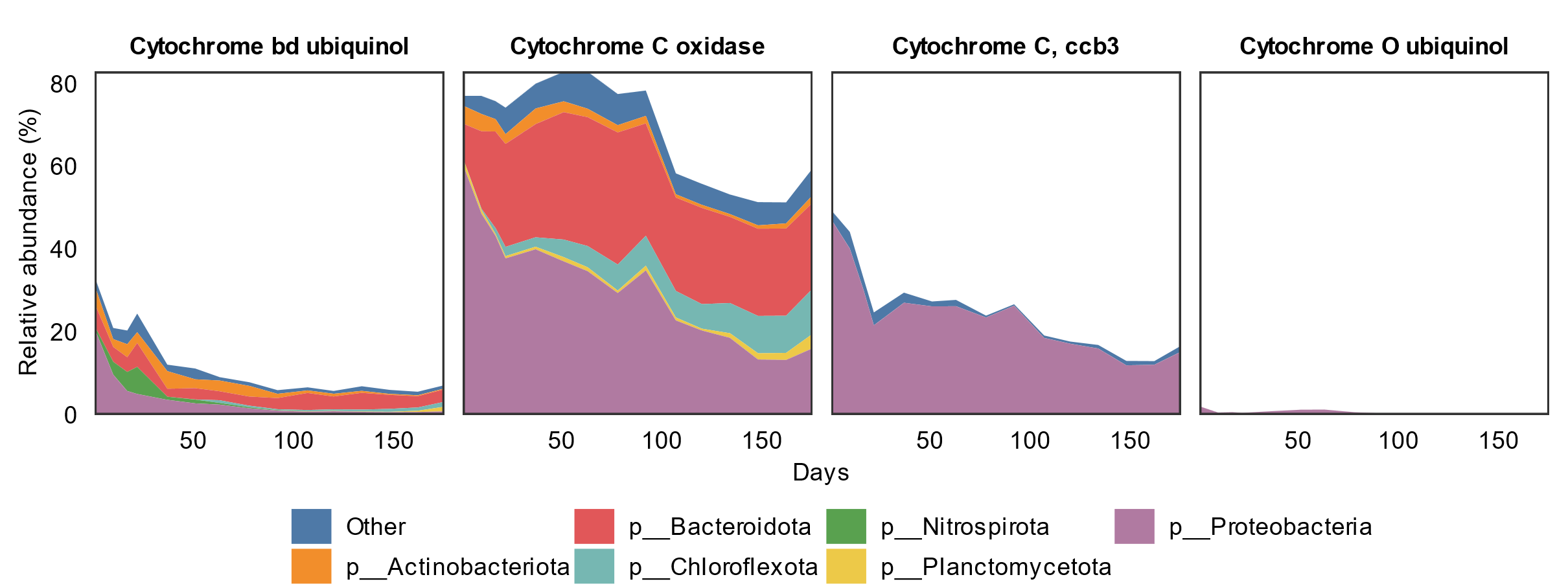


**Figure S2:** Abundance of MAGs with cytochrome oxidases.


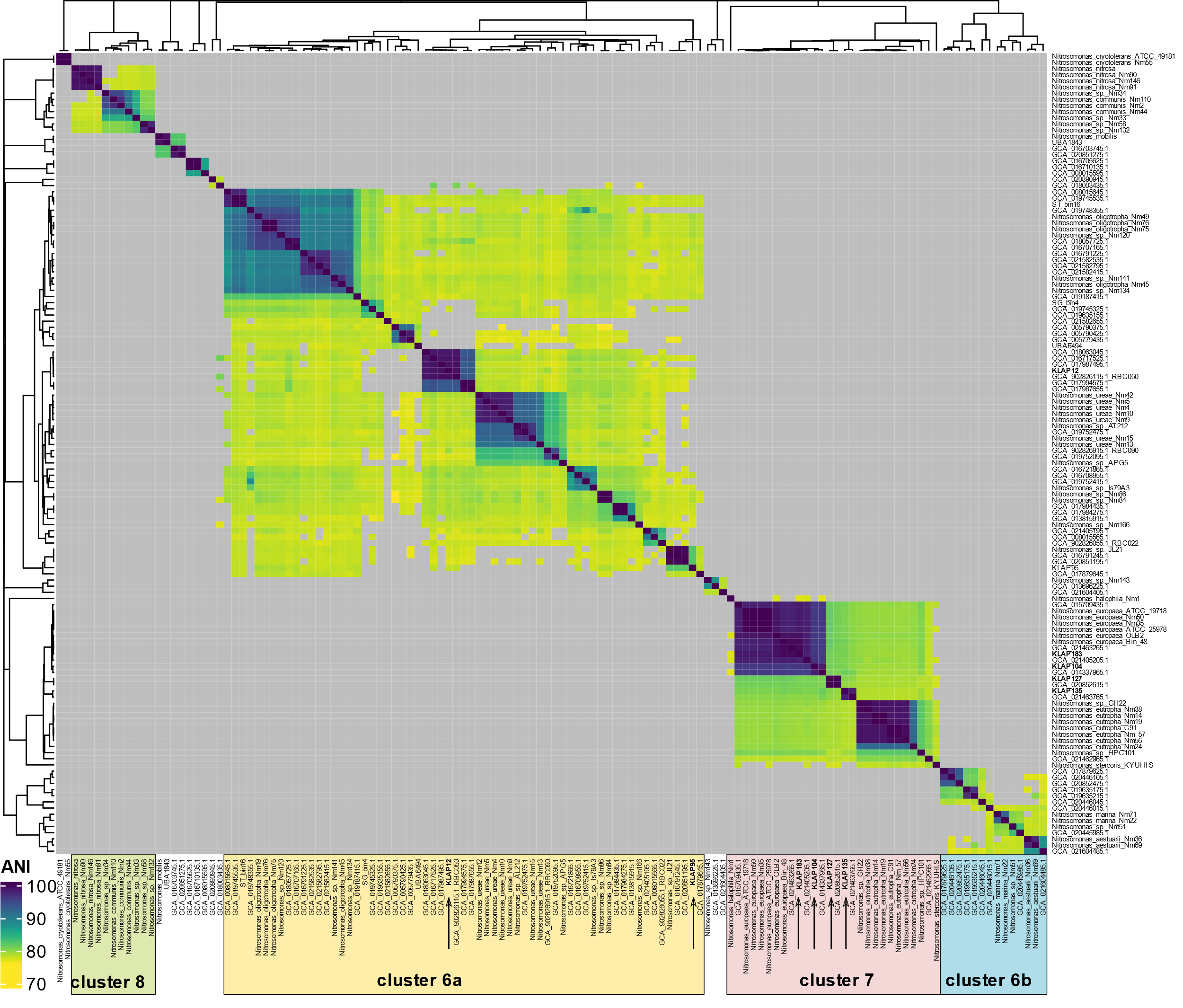


**Figure S3:** Average nucleotide identity of *Nitrosomonas* genomes and MAGs from this study.


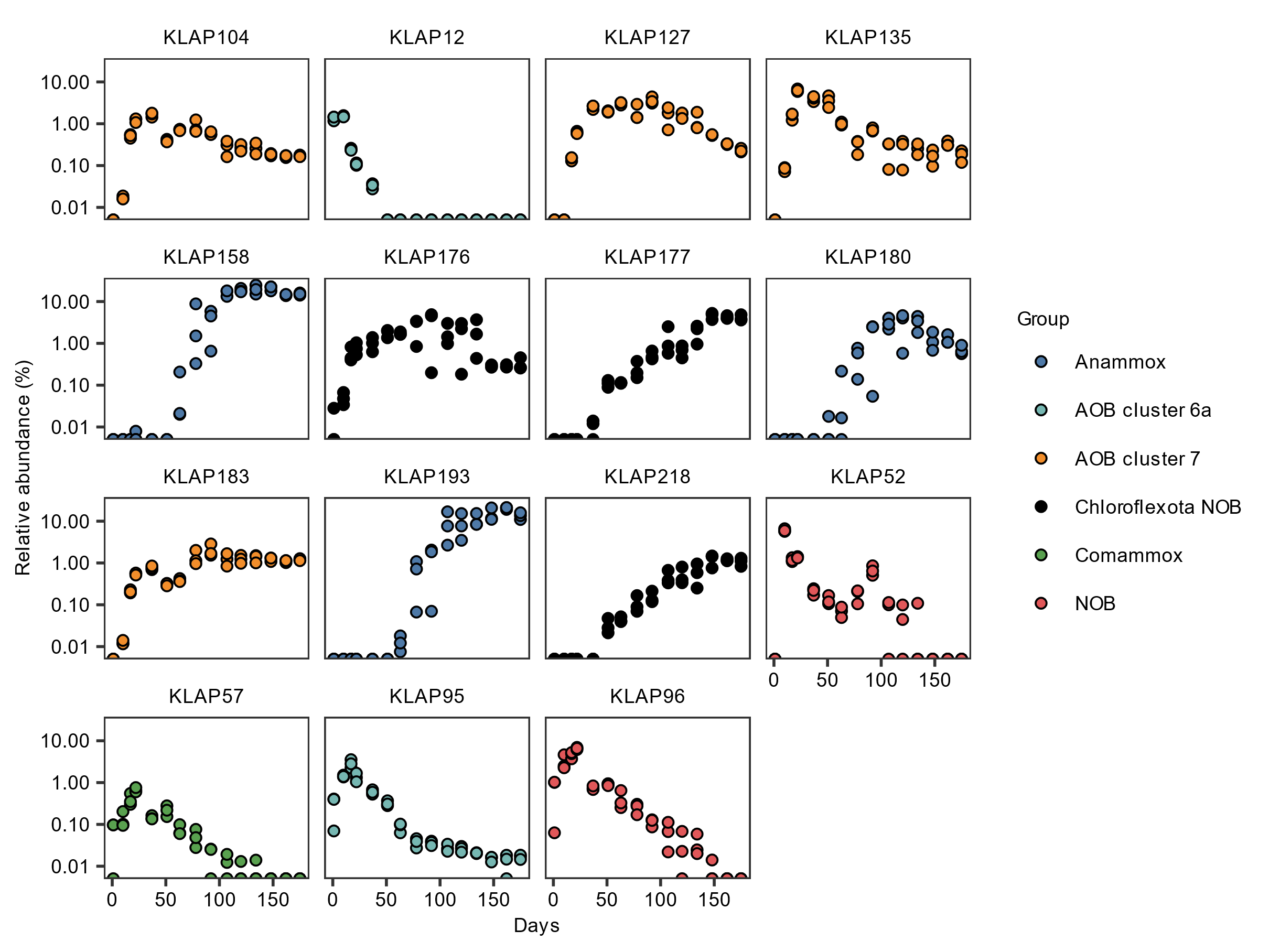


**Figure S4:** Relative abundance of ammonia oxidizers, nitrite oxidizers, putative *Chloroflexota* nitrite oxidizers and anammox bacteria MAGs.


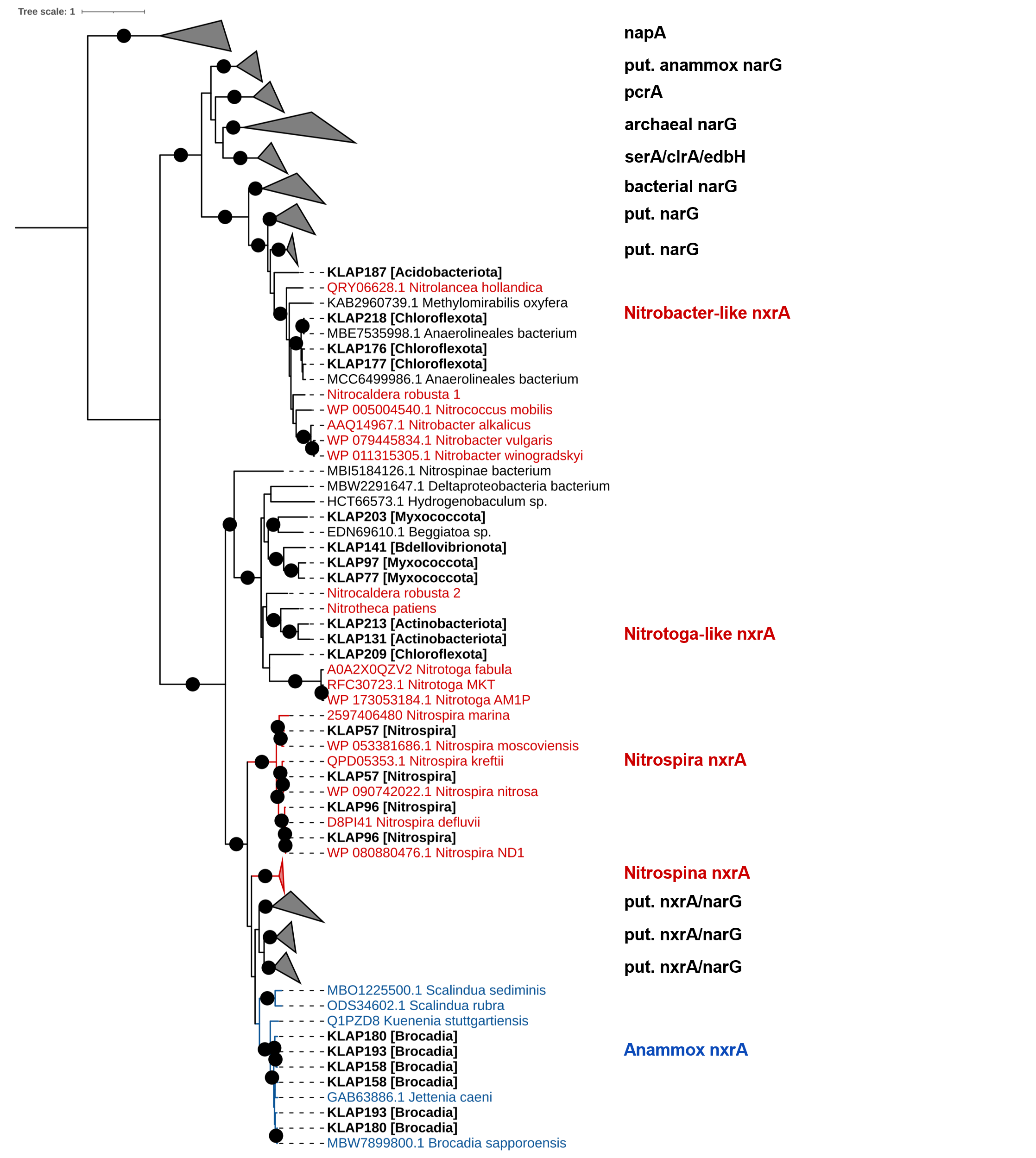


**Figure S5:** Phylogenetic tree of DMSO reductase family type II. Putative NxrA/NarG recovered from MAGs in this study are shown in bold. Red labels are known NOB. Circles show branches with more than 95% support. Periplasmatic nitrate reductase (*napA*) was used as the outgroup. The “put. anammox *narG*” cluster, includes *narG*-like genes similar to the putative *narG* of *Ca*. Jettenia ecosi [16].


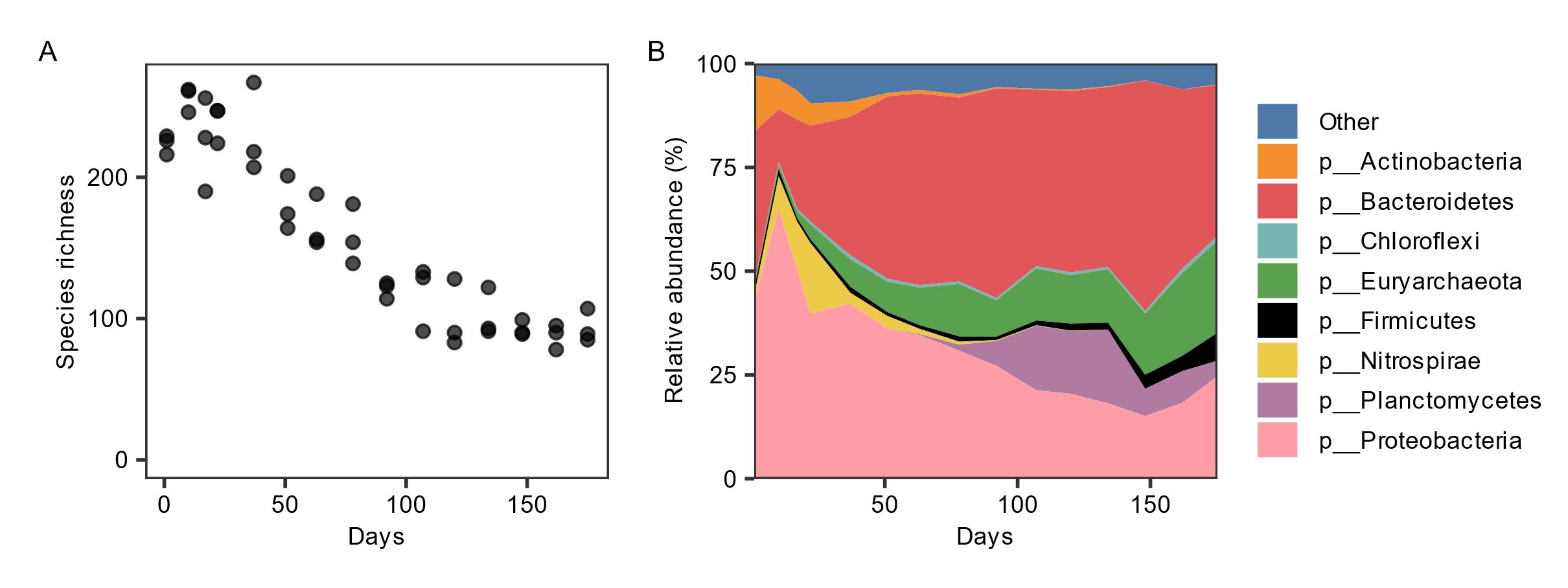


**Figure S6**. Changes in the overall microbial community assessed with Metaphlan. **A:** Changes in species richness. **B:** Changes in relative abundance of major phyla.


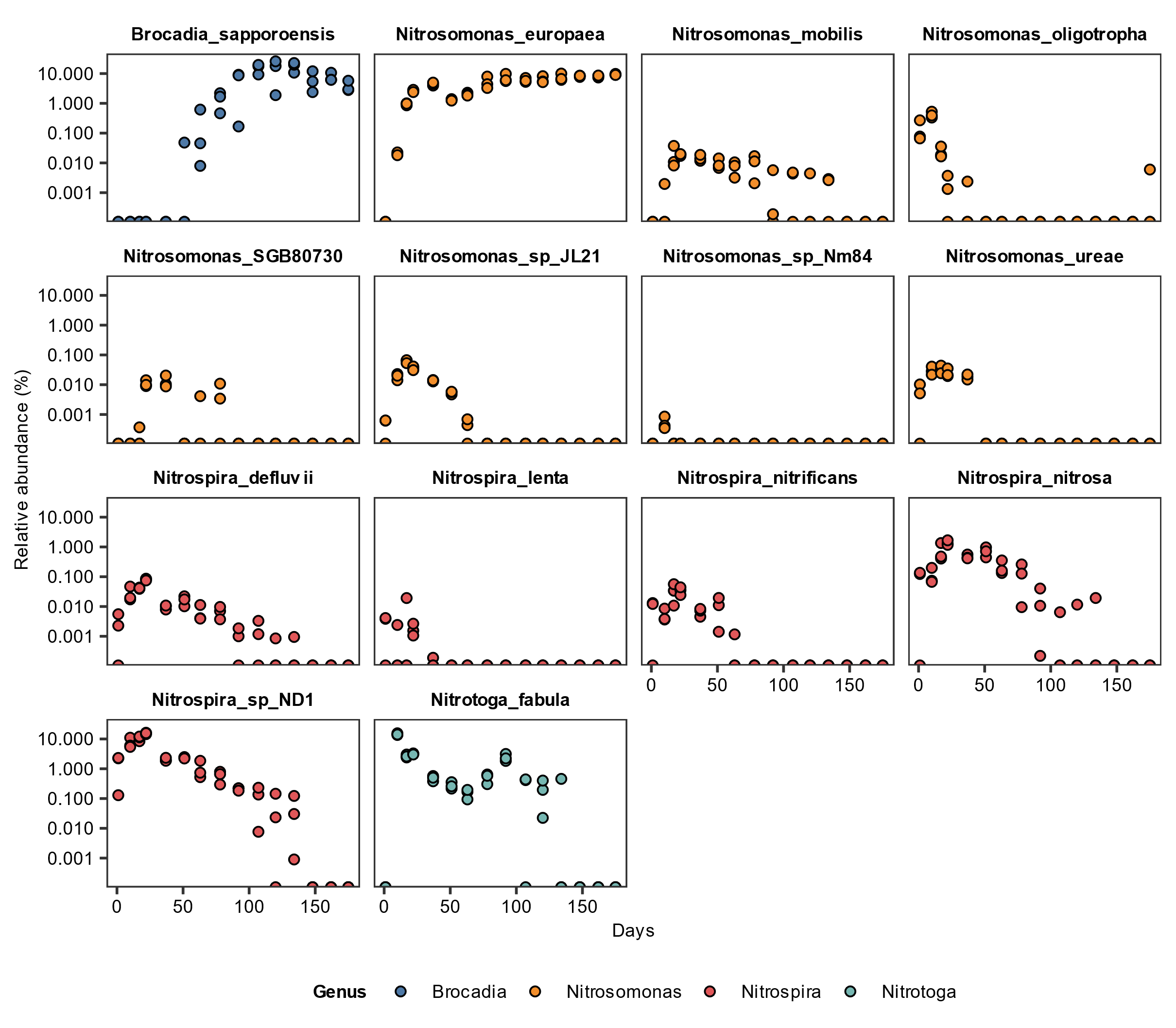


**Figure S7**. Relative abundance of nitrogen converters (nitrifiers, and anammox bacteria) at the species level, as assessed with Methaplan.

# SUPPORTING TABLES

**Table S1:** Pearson correlation values between reactor conditions and phylogenetic diversity for all bacteria. Highly significant values (p<0.001) are marked with an asterisk (*).

|  | SES-MNTD | |  | SES-PD |  |
| --- | --- | --- | --- | --- | --- |
|  | **Correlation** | **p-value** |  | **Correlation** | **p-value** |
| Centrate - Flow(m^3^/h) | -0.06 | 6.89E-01 |  | 0.62 | 5.76E-06* |
| PSW - Flow(m^3^/h) | -0.42 | 4.45E-03 |  | -0.78 | 2.30E-10* |
| Temperature (°C) | -0.18 | 2.24E-01 |  | 0.47 | 1.10E-03 |
| HRT (h) | 0.38 | 9.98E-03 |  | 0.75 | 2.11E-09* |
| DO (mg/l) | -0.3 | 4.81E-02 |  | 0.08 | 6.14E-01 |
| NH_4_^+^ - N(mg/L) | 0.5 | 4.11E-04* |  | -0.07 | 6.32E-01 |
| NH_3_ - N(mg/L) | 0.56 | 6.31E-05* |  | 0.43 | 3.02E-03 |
| NO_2_^-^ - N(mg/L) | 0.54 | 1.36E-04* |  | 0.08 | 6.23E-01 |
| NO_3_^-^ - N(mg/L) | 0.04 | 8.13E-01 |  | 0.07 | 6.69E-01 |

# SUPPORTING METHODS

## Eukaryotic MAGs

Between 0.4% to 14% of the assemblies were classified as eukaryotic with eukrep [1]. Binning was done for each eukaryotic assembly with Metabat2 [2], keeping only bins with more than 10 Mbp. The bins were dereplicated in dRep [3], with a 98% ANI threshold, and skipping the CheckM quality control step; this yielded six MAGs between 15Mb and 31 Mbp.

Gene prediction for these MAGs was performed using MetaEuk [4], with the genetic code for peritrichs [5], and by using the MetaEuk database (2020_TAX_DB) as reference, which is based on the MMETSP [6, 7] and Uniclust90 [8] resources. MetaEuk was also used to assign taxonomy to contigs with MMseqs2 taxonomy [9] by estimating the lowest common ancestor using their 2bLCA approach. Completeness was assessed with BUSCO 5.0.0 [10]. It is unlikely that these were prokaryote composite MAGs, as BUSCO showed low completeness with the bacteria dataset for these MAGs, but when using the *Alveolata* dataset, 50 to 82% completeness was estimated (File S1). In addition, most contigs were classified as *Eukaryota*, with around 50% of those being assigned as *Ciliophora* (File S1).

The presence of eukaryotes is also supported by assembly of the small subunit ribosomal ribonucleic acid (SSU rRNA) sequences using PhyloFlash [11]. Up to 25% of the SSU reads were eukaryotic, with the highest abundance being for *Peritrichia*.

## Identification of putative *Chloroflexota* NOB

To detect putative novel NOB, nitrate reductase subunit alpha (NxrA) were identified with DIAMOND using as reference the NxrA aminoacid sequences from *Nitrolancea hollandica*, *Nitrobacter winogradskyi*, *Nitrospira defluvii* and *Ca*. Nitrotoga fabula. A multiple alignment of these NxrA and sequences references of NxrA and other Type II DMSO reductases was done with MAFFT v7.407 [12] using the FFT-NS-I method. This alignment was then used to build a tree in IQ-TREE v2.0 [13] with an LG+G4 substitution model.

## Estimation of biofilm thickness

One carrier from each sampling timepoint was stored in 50% PBS and ethanol immediately after sampling and stored at -20°C. To estimate biofilm thickness pictures were taken with a DSRL camera at fixed distance between camera and biofilm carriers, with an image resolution of 52.36px/mm. The raw pictures were imported into Fiji v1.54g [14], cropped and exported as tif files. Image segmentation of the biofilms was done in Illastik v1.4.0 using the pixel classification workflow [15]. The biofilm segmentation was used to estimate thickness in Fiji using the Local thickness plugin. Points with zero biofilm thickness were removed, and estimated average thickness were plotted over time.

# SUPPORTING REFERENCES

1. West PT, Probst AJ, Grigoriev IV, Thomas BC, Banfield JF. Genome-reconstruction for eukaryotes from complex natural microbial communities. Genome Res. 2018;28:569–80.

2. Kang DD, Li F, Kirton E, Thomas A, Egan R, An H, et al. MetaBAT 2: an adaptive binning algorithm for robust and efficient genome reconstruction from metagenome assemblies. PeerJ. 2019;7:e7359.

3. Olm MR, Brown CT, Brooks B, Banfield JF. dRep: a tool for fast and accurate genomic comparisons that enables improved genome recovery from metagenomes through de-replication. ISME J. 2017;11:2864–8.

4. Karin EL, Mirdita M, Söding J. MetaEuk—sensitive, high-throughput gene discovery, and annotation for large-scale eukaryotic metagenomics. Microbiome. 2020;8:48.

5. Sánchez-Silva R, Villalobo E, Morin L, Torres A. A New Noncanonical Nuclear Genetic Code: Translation of UAA into Glutamate. Current Biology. 2003;13:442–7.

6. Keeling PJ, Burki F, Wilcox HM, Allam B, Allen EE, Amaral-Zettler LA, et al. The Marine Microbial Eukaryote Transcriptome Sequencing Project (MMETSP): Illuminating the Functional Diversity of Eukaryotic Life in the Oceans through Transcriptome Sequencing. PLOS Biology. 2014;12:e1001889.

7. Johnson LK, Alexander H, Brown CT. Re-assembly, quality evaluation, and annotation of 678 microbial eukaryotic reference transcriptomes. GigaScience. 2019;8.

8. Mirdita M, von den Driesch L, Galiez C, Martin MJ, Söding J, Steinegger M. Uniclust databases of clustered and deeply annotated protein sequences and alignments. Nucleic Acids Research. 2017;45:D170–6.

9. Mirdita M, Steinegger M, Breitwieser F, Söding J, Levy Karin E. Fast and sensitive taxonomic assignment to metagenomic contigs. Bioinformatics. 2021. https://doi.org/10.1093/bioinformatics/btab184.

10. Manni M, Berkeley MR, Seppey M, Simão FA, Zdobnov EM. BUSCO Update: Novel and Streamlined Workflows along with Broader and Deeper Phylogenetic Coverage for Scoring of Eukaryotic, Prokaryotic, and Viral Genomes. Mol Biol Evol. 2021. https://doi.org/10.1093/molbev/msab199.

11. Gruber-Vodicka HR, Seah BKB, Pruesse E. phyloFlash: Rapid Small-Subunit rRNA Profiling and Targeted Assembly from Metagenomes. mSystems. 2020. https://doi.org/10.1128/mSystems.00920-20.

12. Katoh K, Standley DM. MAFFT Multiple Sequence Alignment Software Version 7: Improvements in Performance and Usability. Molecular Biology and Evolution. 2013;30:772–80.

13. Minh BQ, Schmidt HA, Chernomor O, Schrempf D, Woodhams MD, von Haeseler A, et al. IQ-TREE 2: New Models and Efficient Methods for Phylogenetic Inference in the Genomic Era. Molecular Biology and Evolution. 2020;37:1530–4.

14. Schindelin, J., Arganda-Carreras, I., Frise, E. et al. Fiji: an open-source platform for biological-image analysis. Nat Methods. 2012; 9: 676–682. https://doi.org/10.1038/nmeth.2019

15. Berg, S., Kutra, D., Kroeger, T. et al. ilastik: interactive machine learning for (bio)image analysis. Nat Methods. 2019; 16: 1226–1232. https://doi.org/10.1038/s41592-019-0582-9

16. Mardanov AV, Beletsky AV, Ravin NV, Botchkova EA, Litti YV, Nozhevnikova AN. Genome of a Novel Bacterium “Candidatus Jettenia ecosi” Reconstructed From the Metagenome of an Anammox Bioreactor. Front Microbiol. 2019;10:2442.
